# CCD Multi-Ion Image Sensor with Four 128 × 128 Pixels Array

**DOI:** 10.3390/s19071582

**Published:** 2019-04-01

**Authors:** Toshiaki Hattori, Fumihiro Dasai, Hikaru Sato, Ryo Kato, Kazuaki Sawada

**Affiliations:** 1 Department of Electrical and Electronic Information Engineering, Toyohashi University of Technology, Hibarigaoka 1-1, Tenpaku, Toyohashi 441-8580, Japan; zr854452@bf7.so-net.ne.jp (F.D.); hikarusato0824@docomo.ne.jp (H.S.); 2 Cooparative Research Facility Center, Toyohashi University of Technology, Hibarigaoka 1-1, Tenpaku, Toyohashi 441-8580, Japan; ryo_kato@crfc.tut.ac.jp

**Keywords:** CCD ion sensor, multi-ion image, CMOS technology, ink-jet printing, bioactive cations

## Abstract

A semiconductor array pH image sensor consisting of four separated blocks was fabricated using charged coupled device (CCD) and complementary metal oxide semiconductor (CMOS) technologies. The sensing surface of one of the four blocks was Si_3_N_4_ and this block responded to H^+^. The surfaces of the other three blocks were respectively covered with cation sensitive membranes, which were separately printed with plasticized poly (vinyl chloride) solutions including Na^+^, K^+^, and Ca^2+^ ionophores by using an ink-jet printing method. In addition, each block of the image sensor with 128 × 128 pixels could have a calibration curve generated in each independent measurement condition. The present sensor could measure the concentration image of four kinds of ions (H^+^, K^+^, Na ^+^, Ca^2+^) simultaneously at 8.3 frames per second (fps) in separated regions on a chip.

## 1. Introduction

Semiconductor ion sensors are suitable for visualizing local chemical species, because of the capabilities of miniaturization and integration of the sensing area. Many miniaturized sensing pixels based on ISFET were two-dimensionally lined up neatly as an array-imaging sensor [1,2,3,4,5], and a semiconductor-sensing device was two-dimensionally divided into many sensing areas by an addressable light such as LAPS [6,7,8,9,10]. Although almost 50 years has passed since the introduction of the ISFET by Bergveld in 1970 [11], there are still many challenges to be overcome beyond ISFETOLOGY including the REFET subject, in which he summarized the device and readout circuity reported in 2010 [12]. In particular, the array image sensor requires a dense, reliable and scalable ISFET array that enables massive parallelism and high throughput [13]. Recently, several array image sensors were reported including 32 × 32 (1k) pixels and 9.3 frame/s (fps) image sensor [14]; 512 × 576 (300k) and 375 fps image sensor [15]; 3600 × 3600 (13M) and 26 fps image sensor [16]. The number of pixels and fps is increasing, and the spatial resolution is also improving. However, a high operation stability of the array image sensor is required. Therefore, it is important for a reliable array image sensor to demonstrate the image consisting of whole pixels, which indicates the total quality of achievement of the image sensor.

We had developed a different type of semiconductor array pH sensor (liner 8 pixels) based on charged coupled device (CCD) and complementary metal oxide semiconductor (CMOS) technologies in 1999 [17], as an aim at developing the image sensor. According to the plan to finely visualize local chemical species, the CCD pH sensor has steadily progressed to two-dimensional pH image sensors; from the initial stage of 10 × 10 (100) pixels and 30 fps [18]; 32 × 32 (1k) and 5 fps pixels [19]; 128 × 128 and 50 fps (16k) pixels [20]; to 1320 × 976 and 27.5 (1.5M) pixels [21]. The size of sensing pixels became small, and the spatial resolution increased; 100 × 100 μm^2^ [18], 23.55 × 23.55 μm^2^ [19], 12.1 × 12.1 μm^2^ [20], 3.75 × 3.75 μm^2^ [21], respectively. Each image that consisted of whole pixels was demonstrated by an investigation of the sensor response to pH change.

The CCD pH image sensor can also convert into several CCD ion image sensors using the surface coating of ion sensitive membranes. The image sensors monitored local concentrations of metal ions such as K^+^ [22], and biogenic amines [23]. The K^+^ image sensor was applied to a tissue stimulation response of a cultured hippocampal slice stimulated by glutamate [22]. The biogenic amine image sensor was applied to mast cells stimulation response of mast cells by compound 48/80 [23]. On the other hand, living cells have several ion pumps and ion channels for H^+^, Na^+^, K^+^, Ca^2+^, Cl^−^, and so on, to maintain the homeostasis. Therefore, a minute investigation of cell dynamics is required to simultaneously monitor these concentration changes. As the previous studies, we fabricated the CCD multi-ion image sensor for Na^+^-K^+^ [24], K^+^-Ca^2+^ [25]. The K^+^-Ca^2+^ image sensor was applied to the Ca^2+^ release of cultured PC12 cells stimulated by acetylcholine [25]. The multi-ion image sensor was superior to monitor their concentration changes simultaneously for only one experiment.

The multi-image sensors were successfully demonstrated, however, further developments were required; increments of numbers of ions measured simultaneously, clear distinction and refinement of separated sensing regions. For instance, multi-ion-vision equipment that displays a high-resolution image of many ions simultaneously such as a color television is one of the ultimate goals. In this paper, we fabricated a new CCD multi-ion image sensor with four divided regions of 128 × 128 pixels array in order to accomplish the measurement of several ions simultaneously and the clearly separated sensing regions on the chip. Each region of the new image sensor can individually configure the V_ref_. Thus, the sensor can monitor four-ion concentration changes simultaneously. The establishment of the simultaneous monitoring technique for four ions is important in the developing process in order to develop the future multi-ion-vision equipment. The CCD multi-ion image sensor was fabricated from a newly designed CCD pH image sensor with four divided regions of 128 × 128 sensing pixels. Thereafter, three regions were prepared with Na^+^, K^+^, and Ca^2+^ sensitive membranes. These sensitive membranes were constructed by an ink-jet printing method. The present sensor could simultaneously determine the changes of pH, Na^+^, K^+^, and Ca^2+^ concentrations in an intracellular concentration region.

## 2. Fabrication Process and Readout Procedure

### 2.1. Pixel Design and Sensor Chip

**Figure 1:**
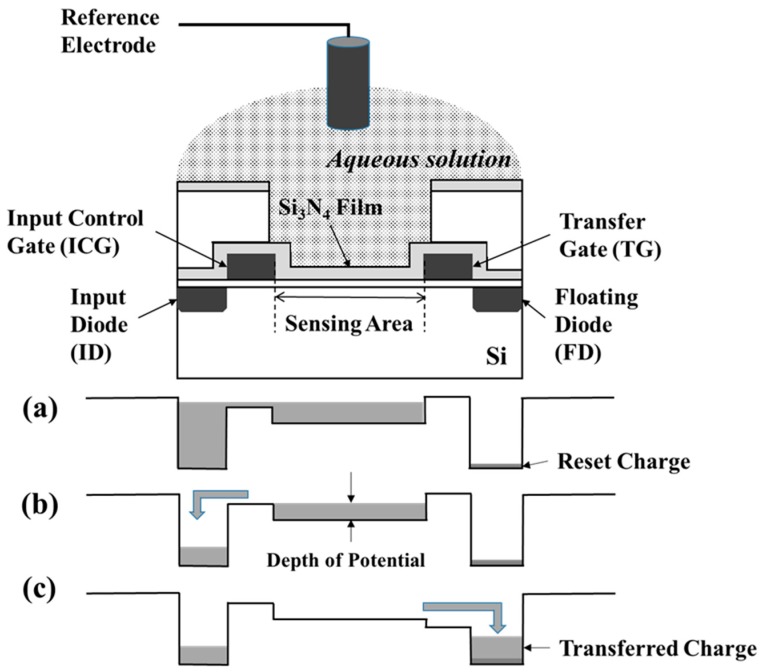
Cross-sectional view of a sensing pixel and measurement principle to focus on charges. (**a**) Charge supply into the depth of potential; (**b**) holding of charges corresponding to the depth; (**c**) transferring of charges to measure.

The sensor system using CMOS technology based on the previous block diagram of 128 × 128 sensor [14] was designed and fabricated. The main circuit design and pixel structure were the same. The present ion image sensor has four times more pixels than the 128 × 128 pixels sensor. Here, the basic sensing principle was briefly mentioned using Figure 1 with the cross-sectional view of a sensing pixel. The key is to be able to measure the surface membrane potential between the aqueous solution and the membrane or the Si_3_N_4_ film in Figure 1. Hydrogen ions adsorb and desorb onto the Si_3_N_4_, and affect the surface membrane potential in the sensing area. On the other hand, when Si_3_N_4_ was covered with a plasticized polyvinyl chloride (PVC) membrane including a cation selective ionophore, the cations adsorb and desorb onto the membrane, and change the surface membrane potential. Thus, their surface membranes depend on the concentration of the sensitive cations. The sensor chip has four terminals to process the charge. The input control gate (ICG) and transfer gate (TG) electrodes are used to control the potential level as the gating. The input diode (ID) and floating diffusion (FD) are pn junctions that supply the charge and detect the quantity of charge, respectively. With a decreasing ID potential, the charge exceeds over ICG, and is filled at the charge sensor area corresponding to the membrane potential, as shown in Figure 1a. With an increasing ID potential, the charge is spilled and holds, as shown in Figure 1b. With an increasing TG, the charge is transferred into FD, as shown in Figure 1c. The charge process can be repeated, and the charge is accumulated without noise. Therefore, the sensitivity is increasing, which is an excellent advantage for the CCD sensor.

**Figure 2:**
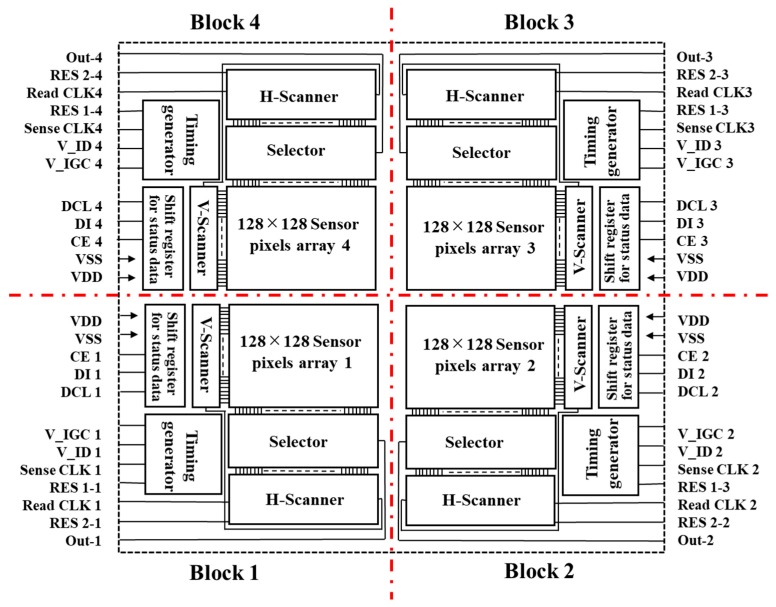
Block design for the present multi-ion sensor.

The structure of the sensor chip and the wiring on the package board is shown in Figure 2. In order to hold a compact size and use previous equipment, the sensor chip is divided into four blocks and each block has a timing generator, shift register, horizontal and vertical scanner, respectively. Each terminal with the same name except for CE is electrically connected on a package board. 

**Figure 3:**
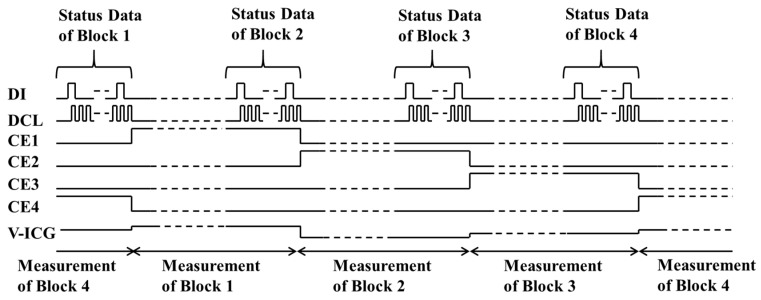
Serial timing chart for the operation of four blocks.

The switching time of each CE terminal is due to the order, as shown in Figure 3. The block whose CE terminal becomes high is activated. The measurement conditions data of each block are saved into Shift resister when CE terminal changes to be high.

**Figure 4:**
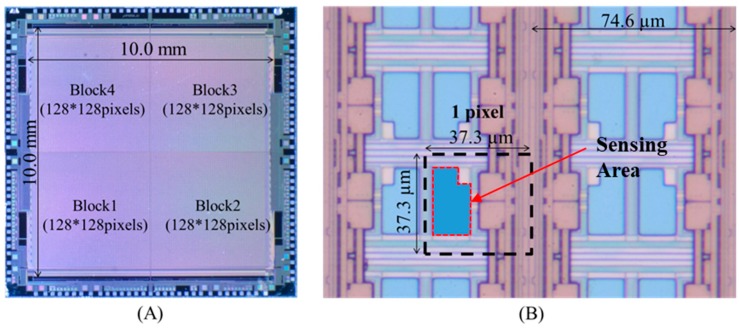
(**A**) A picture of the 256 × 256 array charged couple device (CCD)-type image sensor; (**B**) a micrograph of the sensor pixels.

The whole photographs of a sensor chip and its extended image were shown in Figure 4. The 4 × 16K array CCD ion image sensor possessed 65,536 sensing pixels in a two-dimensional 4 × 128 × 128 array, as shown in Figure 4A. The dimensions of the pixel and the sensor area were 37.3 × 37.3 μm^2^, 9.549 × 9.549 mm^2^, respectively. The interval of blocks was 20 μm. The chip size was 12.2 × 12.2 mm^2^. The frame speed was constant at 8.3 fps. The sensing area of a pixel was in a hollow of ~ 3 µm depth and its size was 13.5 μm × 24.5 μm, as shown in Figure 4B.

### 2.2. Reagents

Bis[(12-crown-4)-methyl]-2-dodecyl-2-methylmalonate (bis(12-crown-4)), 2-Nitrophenyloctyl ether (NPOE), sodium tetrakis[3,5-bis(trifluoromethyl)phenyl]borate (NaTFPB) were purchased from Dojindo Lab., Inc. (Kumamoto, Japan). Polyvinylchloride (MW = 80,000) (PVC), 10,19-Bis [(octadecylcarbamoyl)methoxyacetyl]-1,4,7,13,16-pentaoxa-10,19-diazacycloheneicosane (K23E1), valinomycin, 1,3,5,7,9,11,13,15-oct(propylmethacryl)pentacyclo[9.5.1.13,9.15,15.17,13]octasiloxane (POSS), and tris(hydroxymethyl)aminomethane (Tris) were purchased from Sigma-Aldrich, Inc. (St. Louis, MO, USA). Potassium tetrakis(4-chlorophenyl)borate (K-TCPB) was purchased from Tokyo Chemical Industry (Tokyo, Japan). Dioctyl sebacate (DOS), n-dioctyl phthalate (DOP), tetrahydrofuran (THF), cyclohexanone (CHN), cyclohexane, and other reagents were purchased from Wako Pure Chemical Industries, Ltd. (Osaka, Japan). Water deionized by a Milli-Q system was used in all the experiments. Metal ion solutions were prepared daily by dilution from a 1 M stock solution of each metal chloride.

### 2.3. Ink-Jet Device and the Printing Conditions

An ink-jet apparatus (IJK-200T, Microjet, Shiojiri, Japan) was set up in a large glove box. The nozzle diameter (IJHB-300) was 300 µm. The apparatus was a piezoelectric dot ink-jet printer, and can place the printing solution at the desired point on a chip. The state of the produced drops was monitored using a strobe light camera. The proper adjustment of two voltage pulses on the operating piezoelectric device formed one drop. The pulse conditions were adjusted daily; 80.0–85.0 V as the pulse voltages, 5.0–10.0 µS as the 1st pulse range, 3.0–10.0 µS as the interval pulse range, 3.0–10.0 µS as the 2nd pulse range. One drop of the cocktail solution covered about 36 (6 × 6) sensing pixels. The rate of line printing and the interval of the pitch decided in each ion sensitive membranes. The waiting time before the recoating was 10 s. After printing, the membrane was dried for 24 h at room temperature. Since the ink-jet apparatus had only a single injection head, when a multi-ion membrane was prepared, each solution was separately printed.

### 2.4. Procedure of the Preparation of Each Sensitive Membrane

In order to increase the adhesion of the plasticized PVC membrane on the chip, the ink-jet printing areas were pretreated with sodium hydroxide solution. A 0.1 mM sodium hydroxide solution laid on the ink-jet printing area for more than 30 min, and washed with pure water, and the sensor was dried in a clean glove box at room temperature. After the pretreatment, each membrane was formed by the ink-jet method. For the Na^+^ sensitive printing solution, PVC 44.0 mg, POSS 30.0 mg, DOP 22.0 mg, Bis (12-crown-4) 2.7 mg, TFPB 1.3 mg were dissolved in a solvent mixture of 5 mL of THF and 5 mL of CHN (THF-CHN solution). The mixed printing solution was dropped into the Na^+^ sensing area with a number of overcoats of 20 times. At this time, the pitch of the drop was set to 100 μm. For the K^+^ sensitive printing solution, PVC 30.0 mg, POSS 30.0 mg, DOS 36.0 mg, valinomycin 2.7 mg, K-TCPB 1.3 mg were dissolved in THF-CHN solution of 10 mL. The mixed printing solution was dropped into the K^+^ sensing area with a number of overcoats of 20 times, and the pitch of the drop was set to 80 μm. For the Ca^2+^ sensitive printing solution, PVC 30.0 mg, POSS 11.2 mg, NPOE 55.2 mg, K23E1 3.0 mg, TFPB 1.3 mg were dissolved in THF-CHN solution of 10 mL. The mixed printing solution was dropped into the Ca^2+^ sensing area with the number of overcoats of 40 times, and the pitch of the drop was set to 80 μm.

### 2.5. Measurement of Ion Concentration

The electrochemical cell consisted of a LF-1/2 leak-less Ag/AgCl reference electrode sample solution/plasticized PVC membrane/semiconductor sensor. The potential slopes of sodium ion brock, potassium ion block, and calcium ion block were evaluated from three different concentrations of each ion solutions: 0.1 mol/L (M) Tris-buffered (pH 7.4) solutions with concentrations of 200, 20, and 2 mM sodium ions. 0.1 mol/L (M) Tris-buffered (pH 7.4) solutions with concentrations of 40, 4, and 0.4 mM potassium ions. 0.1 mol/L (M) Tris-buffered (pH 7.4) solutions with concentrations of 18, 1.8, and 0.18 mM sodium ions. These solutions with the calibration were corresponding to the range of extra-cellular concentration [26]. All signals of the sensing pixels were recorded as a video after each pixel arrayed on the chip was calibrated with the above three solutions.

## 3. Results and Discussion

### 3.1. Evaluation of Potential Response to Each Cation

**Figure 5:**
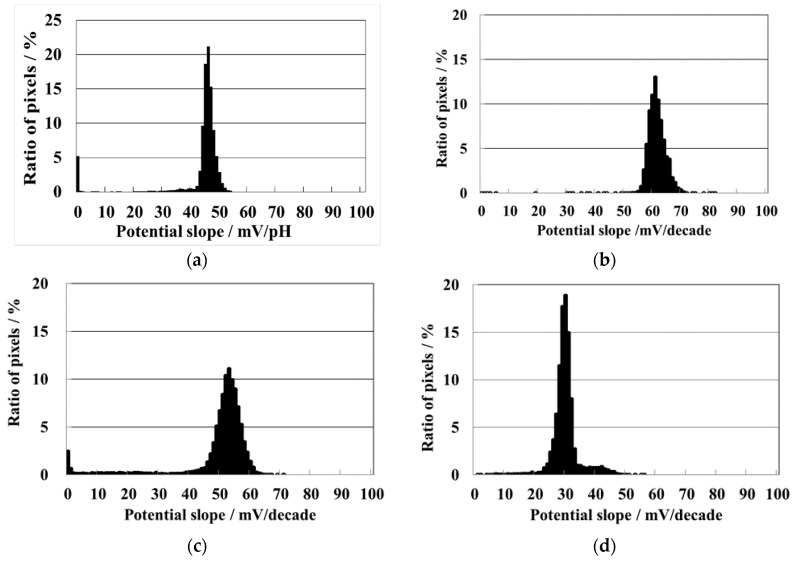
Histograms of potential slopes to (**a**) pH for the rigth upper block; (**b**) sodium ion for the left upper block; (**c**) potassium ion for the right under block; (**d**) calcium ion for the left under block.

The potential response of each block to the corresponding cation was shown in Figure 5a–d. To measure the hydrogen ion sensitivity of the sensor, ordinal buffered pH standard solutions (pH 4, pH 7, and pH 9) were used. The numbers of the right upper block to pH sensing pixels was 16,384. The potential slope showed a distribution with an ideal high kurtosis. The maximum of the frequency distribution of the pH sensitivity was 46 mV/pH (48 mV/pH to pH 4–7, 43 mV/pH to pH 7–9). Although the slope is inferior to Nernstian response, most of the slopes indicated were highly sensitive. The other three blocks were treated with the ink-jet printing. The left upper block of the chip was printed with the sodium ion membrane, and the left under block with the calcium ion membrane, the right under block with the potassium ion membrane. The potential slope of sodium ion block is high and the maximum distribution was 61 [mV/decade] (63 mV/decade to 0.4 mM–4 mM, 60 mV/decade to 4 mM–40 mM) that were almost Nernstian response, as shown in Figure 5b. The frequency distribution has a high kurtosis with a deviation to high value. The potential slope of the potassium ion block is lower than that of the sodium ion block, as shown in Figure 5c. The maximum distribution potential slope was 52 [mV/decade] (53 mV/decade to 2 mM–20 mM, 51 mV/decade to pH 20 mM–200 mM), and has a wide normal distribution. Although many potential responses did not achieve Nernstian slope, the sensitivity was higher than the pH block. The potential slope of calcium ion block is lower than the other cation, as shown in Figure 5d. The lower sensitivity was due to the ion valence of +2. The maximum distribution potential slope was 29.0 [mV/decade] (26 mV/decade to 0.18 mM–1.8 mM, 32 mV/decade to 1.8 mM–18 mM) that was Nernstian slope. The frequency distribution has a high kurtosis and a slight distribution with a small deviation to high value. These potential slopes were not ideal, however, the response was close to Nernstian response or sufficient response.

### 3.2. Dynamic Response

**Figure 6:**
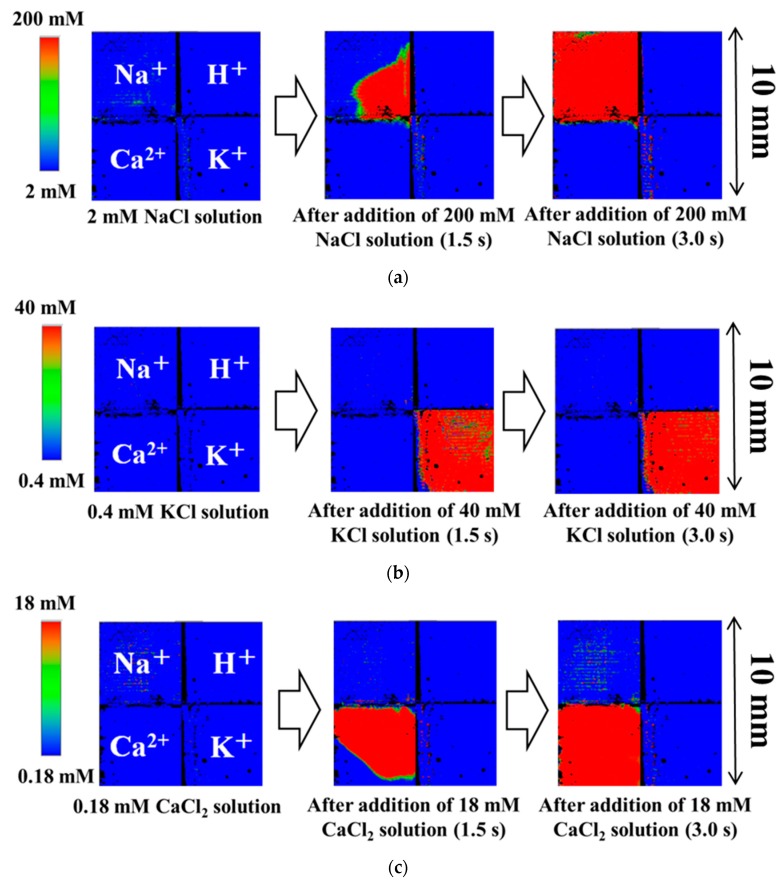
Snapshots of CCD multi-ion image sensor with 128 × 128 pixels array. (**a**) 200 mM sodium ion solution (100 µL) was injected into a 400 µL of sample solution including 2 mM sodium ion solution by a pipette; (**b**) 40 mM potassium ion solution (100 µL) was injected into a 400 µL of sample solution including 0.4 mM potassium ion solution by a pipette; (**c**) 18 mM calcium ion solution (100 µL) was injected into a 400 µL of sample solution including 0.18 mM calcium ion solution by a pipette.

Snapshots of the multi-ion image sensor are shown in Figure 6. When 400 µL of the 200 mM sodium ion solution was added into 400 µL of 2 mM sodium ion solution by a 1000 µL pipette, the color of the left upper block immediately changed from blue to red (Figure 6a). The left upper block responded to the concentration change of sodium ions, but no change appeared in the other block, except for slight areas at the upper edge of calcium ion block and the right edge of potassium ion block. When the sodium membrane was prepared by the ink-jet method, it seemed that the sodium membrane painting solution was leaked to these areas. When 400 µL of the 0.18 mM calcium ion solution was added into 400 µL of 18 mM calcium ion solution by a 1000 µL pipette, the color of the left under block immediately changed from blue to red (Figure 6b). The left under block responded to the concentration change of calcium ions, but no change appeared in the other block. When 400 µL of the 0.4 mM potassium ion solution was added into 400 µL of 40 mM potassium ion solution by a 1000 µL pipette, the color of the right under block immediately changed from blue to red (Figure 6c). The right under block responded to the concentration change of potassium ions, but no change appeared in the other block, except for the sodium ion block. The sodium ion block had a slightly potential response to calcium ion concentration. All the concentration changes were completed in three s. For all additions of the pipette, the pH region of the upper right did not show a concentration change. This is very effective to selectively detect ion behaviors based on cell functions without considering pH variation.

**Figure 7:**
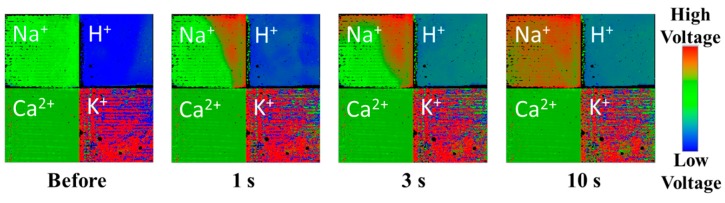
Visual image changes for the instant addition of a phosphate buffer solution (pH 7) to a borate buffer solution (pH 9). This sensor chip is different from the sensor chip shown in Figure 6.

In order to demonstrate the visualization of the mixture solution, a phosphate buffer solution of 100 μL (pH 6.86, 0.025 M di-hydrogen potassium phosphate +0.025 M hydrogen di-sodium phosphate) was added to a borate buffer solution of 400 μL (pH 9.18, 0.01 M borate solution of half-neutralization by NaOH), as shown in Figure 7. The color of the Na^+^ region gradually changed because of increasing Na^+^ concertation from 0.005 M to 0.01 M. The color of the H^+^ quickly changed because of pH 9 to about 7. The color of part of the K^+^ region changed because of increasing K^+^ concentration to 0.005 M. Unfortunately, the preparation of K^+^ membrane of this chip was insufficient. Since the two solutions did not contain Ca^2+^, no color change was observed in the Ca^2+^ region. The change of these colors was reasonable so that the simultaneous visualization by the change of three ions was demonstrated.

## 4. Conclusions

We fabricated a CCD ion multi-image sensor that has a sodium ion region, a potassium ion region, a calcium ion region, and a hydrogen ion region. Each region except for the hydrogen ion region was respectively covered with the cation sensitive membrane, which was formed on the chip by an ink-jet method. Each sensitive block sufficiently responded to the corresponding cation. In addition, we were able to observe a dynamic change of ion concentration as an animation. These results showed that this sensor didn’t have any problem as the structure of the multi-ion sensor essentially. This type of sensor will be one of the useful tools for biochemical application. The subjects of increments of numbers of ions measured simultaneously, and the distinction of separated sensing regions was accomplished. The concentration of the measurable cations is relatively high at the biological environment. Therefore, the sensitivity, which is the ability to identify the difference between large concentrations, rather than the detection limit is required. The present sensor can demonstrate the difference of the Ca^2+^ release of cultured PC12 cells stimulated by acetylcholine at least, because of the same basic design as the Ca^2+^-K^+^ sensor [25]. Moreover, the increment of accumulation measurable cycles by a function which was furnished in the present sensor system can further reduce the noise and improve the sensitivity to be able to distinct the concentration of less than 0.01 of pH, or –log [M] [27]. Except for milli-sec order study such as neural cells, sec order change of cells and tissue can be visualized. However, our CCD sensors are still in the developing stage for the future multi-ion-vision equipment, four blocks were large on the present sensor. Furthermore, refinement of sensing regions is required. Therefore, we are now planning to develop the multi-ion sensor which has small (<30 μm) and numerous ion sensing regions which are uniformly placed in the whole chip. On the other hand, improvement of the frame rate of the imaging is also required. The present sensor system has only one single analog to digital converter (ADC), so the frame rate is reduced by increasing the numbers of sensing pixels. Therefore, we will be able to improve the frame rate by increasing with the number of ADC.

## References

[1] Meyer H., Drewer H., Krause J., Cammann K., Kakerow R., Manoli Y., Mokwa W., Rosrert M. (1994). Chemical and biochemical sensor array for two-dimensional imaging of anlyte distributions. Sens. Actuators B Chem..

[2] Kakerow R., Manoli Y., Moka W., Rosrert M., Rospert M., Meyer H., Drewer H., Krause J., Cammann K. (1994). A monolithic sensor array of individually addressable microelectrodes. Sens. Actuators A Phys..

[3] Nakazato K. (2009). An integrated ISFET sensor array. Sensors.

[4] Nemeth B., Piechocinski M.S., Cumming D.R.S. (2012). High-resolution real-time ion-camera system using a CMOS-based chemical sensor array for proton imaging. Sens. Actuators B Chem..

[5] Machida S., Shimada H., Motoyama Y. (2018). Multiple-channel detection of cellular activities by ion-sensitive transistors. Jpn. J. Appl. Phys..

[6] Hafeman D.G., Parce J.W., McConnell H.M. (1988). Light-addressable potentiometric sensor for biochemical systems. Science.

[7] Qintao Z., Ping W., Parak W.J., George M., Zhang G. (2001). A novel design of multi-light LAPS based on digital compensation of frequency domain. Sens Actuators B Chem..

[8] Yoshinobu T., Schöning M.J., Finger F., Moritz W., Iwasaki H. (2004). Fabrication of thin-film LAPS with amorphous silicon. Sensors.

[9] Guo Y., Seki K., Miyamoto K., Wagner T., Schöning M.J., Yoshinobu T. (2014). Novel photoexcitation method for light-addressable potentiometric sensor with higher spatial resolution. Appl. Phys. Exp..

[10] Wang J., Du L., Krause S., Wu C., Wang P. (2018). Surface modification and construction of LAPS towards biosensing applications. Sens. Actuators B Chem..

[11] Bergveld P. (1970). Development of an ion-sensitive solid-state device for neurophysiological measurements. IEEE Trans. Biomed. Eng..

[12] Bergveld P. (2003). Thirty years of ISFETOLOGY: What happened in the past 30 years and what may happen in the next 30 years. Sens Actuators B Chem..

[13] Moser N., Lande T.S., Toumazou C., Georgiou P. (2016). ISFETs in CMOS and emergent trends in instrumentation: A review. IEEE Sens. J..

[14] Hu Y., Moser N., Georgiou P. (2017). A 32 × 32 ISFET Chemical Sensing Array With Integrated Trapped Charge and Gain Compensation. IEEE Sens. J..

[15] Jiang Y., Liu X., Huang X., Guo J., Yan M., Yu H., Huang J.-C., Hsieh C.-H., Chen T.-T. A 201 mV/pH, 375 fps and 512×576 CMOS ISFET sensor in 65nm CMOS technology. Proceedings of the IEEE Custom Integrated Circuits Conference (CICC).

[16] Cong Y., Xu M., Zhao D., Wu D. A 3600 × 3600 large-scale ISFET sensor array for high-throughput pH sensing. Proceedings of the IEEE 12th International Conference on ASIC (ASICON).

[17] Sawada K., Mimura S., Tomita K., Nakanishi T., Tanabe H., Ishida M., Ando T. (1999). Novel CCD-based pH imaging sensor. IEEE Trans. Electron. Devices.

[18] Hizawa T., Sawada K., Takao H., Ishida M. (2006). Fabrication of a two-dimensional pH image sensor using a charge transfer technique. Sens. Actuators B Chem..

[19] Hizawa T., Matsuo J., Ishida T., Takao H., Abe H., Sawada K., Ishida M. 32 × 32 pH image sensors for real time observation of biochemical phenomena. Proceedings of the International Solid-State Sensors, Actuators and Microsystems Conference.

[20] Futagawa M., Suzuki D., Otake R., Dasai F., Ishida M., Sawada K. (2013). Fabrication of a 128 × 128 Pixels Charge Transfer Type Hydrogen Ion Image Sensor. IEEE Trans. Electron. Dev..

[21] Edo Y., Tamai Y., Yamazaki S., Inoue Y., Kanazawa Y., Nakashima Y., Yoshida T., Arakawa T., Saitoh S., Maegawa M. 1.3 Mega pixels CCD pH imaging sensor with 3.75 μm spatial resolution. Proceedings of the IEEE International Electron Devices Meeting (IEDM).

[22] Kono A., Sakurai T., Hattori T., Okumura K., Ishida M., Sawada K. (2014). Label free bio image sensor for real time monitoring of potassium ion released from hippocampal slices. Sens. Actuators B Chem..

[23] Hattori T., Tamamura Y., Tokunaga K., Sakurai T., Kato R., Sawada K. (2014). Two-dimensional microchemical observation of mast cell biogenic amine release as monitored by a 128 × 128 array-type charge-coupled device ion image sensor. Anal. Chem..

[24] Hattori T., Satou H., Tokunaga K., Kato R., Sawada K. (2015). 16K Array Charge Coupled Device Multi-Ion Image Sensors for Simultaneous Determination of Distributions of Sodium and Potassium Ions. Sens. Mater..

[25] Matsuba S., Kato R., Okumura K., Sawada K., Hattori T. (2018). Extracellular Bio-imaging of Acetylcholine-stimulated PC12 Cells Using a Calcium and Potassium Multi-ion Image Sensor. Anal. Sci..

[26] Lodish H., Berk A., Kaiser C.A., Krieger M., Bretscher A., Ploegh H., Amon A., Martin K. (2016). Molecular Biology of the Cells.

[27] Watanabe E., Hizawa T., Mimura T., Ishida T., Takao K., Sawada K., Ishida M. Low-noise operation of charge-transfer-type ph sensor using charge accumulation technique. Proceedings of the 11th International Conference on Miniaturized Systems for Chemistry and Life Sciences.

